# COVID‐19 metabolism: Mechanisms and therapeutic targets

**DOI:** 10.1002/mco2.157

**Published:** 2022-08-09

**Authors:** Tianshi Wang, Ying Cao, Haiyan Zhang, Zihao Wang, Cheuk Him Man, Yunfan Yang, Lingchao Chen, Shuangnian Xu, Xiaojing Yan, Quan Zheng, Yi‐Ping Wang

**Affiliations:** ^1^ Shanghai Key Laboratory for Tumor Microenvironment and Inflammation Department of Biochemistry and Molecular Cell Biology Shanghai Jiao Tong University School of Medicine Shanghai China; ^2^ State Key Laboratory of Oncogenes and Related Genes Shanghai Cancer Institute Renji Hospital Shanghai Jiao Tong University School of Medicine Shanghai China; ^3^ Bai Jia Obstetrics and Gynecology Hospital Shanghai China; ^4^ Fudan University Shanghai Cancer Center Key Laboratory of Breast Cancer in Shanghai Shanghai Key Laboratory of Radiation Oncology Cancer Institute and The Shanghai Key Laboratory of Medical Epigenetics Institutes of Biomedical Sciences Shanghai Medical College Fudan University Shanghai China; ^5^ Department of Oncology Shanghai Medical College Fudan University Shanghai China; ^6^ The International Co‐laboratory of Medical Epigenetics and Metabolism Ministry of Science and Technology Shanghai China; ^7^ Division of Hematology Department of Medicine University of Hong Kong Pokfulam Hong Kong, China; ^8^ Department of Cell Biology School of Basic Medical Sciences Cheeloo College of Medicine Shandong University Jinan China; ^9^ Department of Neurosurgery Huashan Hospital Shanghai Medical College Fudan University National Center for Neurological Disorders Shanghai Key Laboratory of Brain Function and Restoration and Neural Regeneration Neurosurgical Institute of Fudan University Shanghai Clinical Medical Center of Neurosurgery Shanghai China; ^10^ Department of Hematology Southwest Hospital Army Medical University Chongqing China; ^11^ Department of Hematology The First Affiliated Hospital of China Medical University Shenyang China; ^12^ Center for Single‐Cell Omics School of Public Health Shanghai Jiao Tong University School of Medicine Shanghai China

**Keywords:** antiviral response, metabolic reprogramming, mitochondria metabolism, posttranslational modification, SARS‐CoV‐2

## Abstract

Severe acute respiratory syndrome coronavirus 2 (SARS‐CoV‐2) dysregulates antiviral signaling, immune response, and cell metabolism in human body. Viral genome and proteins hijack host metabolic network to support viral biogenesis and propagation. However, the regulatory mechanism of SARS‐CoV‐2‐induced metabolic dysfunction has not been elucidated until recently. Multiomic studies of coronavirus disease 2019 (COVID‐19) revealed an intensive interaction between host metabolic regulators and viral proteins. SARS‐CoV‐2 deregulated cellular metabolism in blood, intestine, liver, pancreas, fat, and immune cells. Host metabolism supported almost every stage of viral lifecycle. Strikingly, viral proteins were found to interact with metabolic enzymes in different cellular compartments. Biochemical and genetic assays also identified key regulatory nodes and metabolic dependencies of viral replication. Of note, cholesterol metabolism, lipid metabolism, and glucose metabolism are broadly involved in viral lifecycle. Here, we summarized the current understanding of the hallmarks of COVID‐19 metabolism. SARS‐CoV‐2 infection remodels host cell metabolism, which in turn modulates viral biogenesis and replication. Remodeling of host metabolism creates metabolic vulnerability of SARS‐CoV‐2 replication, which could be explored to uncover new therapeutic targets. The efficacy of metabolic inhibitors against COVID‐19 is under investigation in several clinical trials. Ultimately, the knowledge of SARS‐CoV‐2‐induced metabolic reprogramming would accelerate drug repurposing or screening to combat the COVID‐19 pandemic.

## Graphical abstract

SARS‐CoV‐2 deregulates cell metabolism in multiple organs. The interaction between SARS‐CoV‐2 and human hosts remodels metabolic network to regulate viral infection and replication. Metabolism‐based therapy holds promise in the combat against SARS‐CoV‐2.

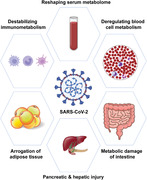

## 1 INTRODUCTION

The coronavirus disease 2019 (COVID‐19) pandemic has become the biggest global health crisis since the beginning of the 21st century.^1^ To date, this villainous virus has swiped over 110 countries and caused millions of deaths.^2^, ^3^ Public health interventions such as immunization and social distancing remain the most effective countermeasure to severe acute respiratory syndrome coronavirus 2 (SARS‐CoV‐2) transmission.^4^ Progress in the clinical treatment of COVID‐19 patients remains dismal. Compounds that suppressed viral infection in vitro turned out to be mostly ineffective in clinical treatment of COVID‐19.^5^, ^6^ Monoclonal antibodies were selectively used to treat high‐risk patients due to a high cost.^7^, ^8^ Of note, nucleotide analogs such as remdesivir displayed broad activity against the circulating variants.^9^ Nirmatrelvir, a compound targeting viral protease to restrict viral replication, was clinically used to treat mild‐to‐moderate COVID‐19 patients.^10^ Nirmatrelvir remains active against Omicron variant.^11^ Additionally, engineered natural killer cells are emerging as immunotherapeutic tools against COVID‐19.^12^ Elucidation of COVID‐19 pathogenesis is essential for discovering new therapeutic opportunities to combat SARS‐CoV‐2.

SARS‐CoV‐2 is a single‐stranded RNA virus that targets multiple tissues in human body. Lung epithelium infection by SARS‐CoV‐2 causes life‐threatening pneumonia. SARS‐CoV‐2 virions are encapsulated in lipid bilayer. The viral genome encodes at least 14 open reading frames (ORFs), which give rise to 29 different proteins.^13^, ^14^ The products encoded by viral genome allow SARS‐CoV‐2 to dysregulate antiviral signaling, immune response, and cell metabolism in human body.

Of note, the SARS‐CoV‐2 genome does not encode metabolic enzymes required for viral genomic replication, protein synthesis, and lipogenesis. To replicate itself, SARS‐CoV‐2 hijacks the metabolic network and biogenesis programs in host cells. For example, SARS‐CoV‐2 boosted lipid biosynthesis to support the generation of lipid bilayer‐enveloped virions in a fashion similar to other coronaviruses.^15^, ^16^ Accordingly, SARS‐CoV‐2 infection causes systemic metabolic changes in the human body. In recent years, multiomics profiling of clinical samples from COVID‐19 patients has revealed key metabolic features of SARS‐CoV‐2 biology. The metabolic network of host cells interacted with the viral genome and its products to regulate viral replication and propagation. Viral hijacking of host cells also generated metabolic vulnerabilities of SARS‐CoV‐2 replication, which could be explored to develop new therapies for SARS‐CoV‐2 infection. Here, we reviewed recent advances in the metabolic interaction between SARS‐CoV‐2 and human body.

## 2 METABOLIC FEATURES OF COVID‐19 PATIENTS

**FIGURE 1:**
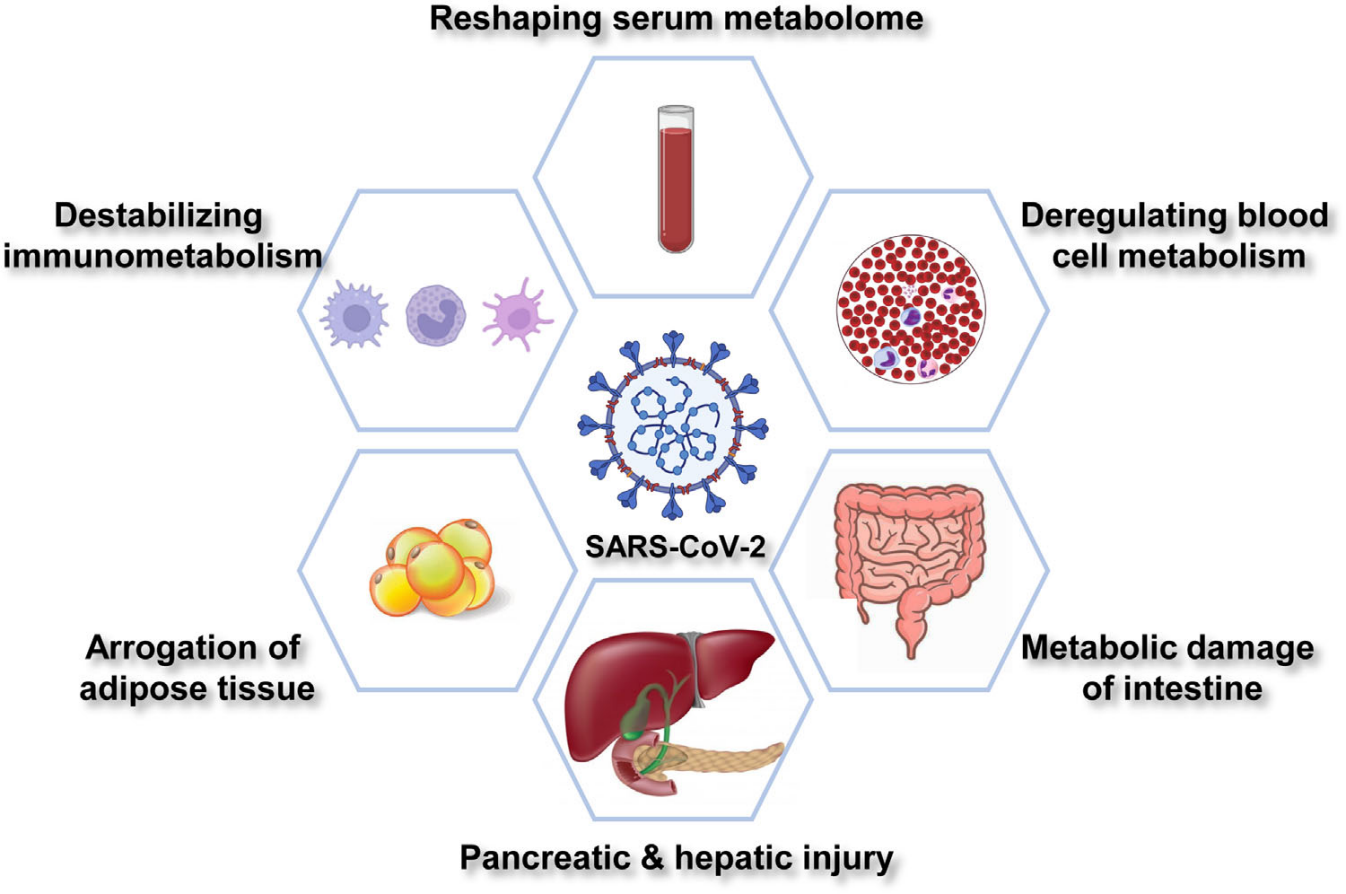
Metabolic features of COVID‐19. SARS‐CoV‐2 infection mediates systemic alterations in host metabolism. COVID‐19 is featured by dysregulated metabolism in serum, blood cells, intestine, pancreas, liver, adipose tissue, and immune system.

SARS‐CoV‐2 infection causes systemic changes at transcriptomic, proteomic, and metabolomic levels. Multiomic profiling becomes a powerful tool in elucidating the metabolic impact of SARS‐CoV‐2 at different stages of infection.^17^ To date, SARS‐CoV‐2 has been shown to dysregulate host metabolism in multiple tissues and organs (Figure 1).

### 2.1 Reshaping serum metabolome

Serum samples are easily accessible from COVID‐19 patients. Alterations of serum metabolites provide mechanistic insights into viral infection‐induced metabolic reprogramming. Recent metabolomic profiling has revealed dramatic changes in glucose, amino acids, and lipid metabolism in COVID‐19 serum (Figure 1).

Dysregulation of glucose metabolism is a key feature as determined by plasma metabolome. COVID‐19 patients generally exhibited an elevation of serum glucose levels, which was in line with an upregulation of glycolytic intermediates.^18^ Glucose metabolism supports the tricarboxylic acid cycle in the mitochondria and produces malate, an indicator of mitochondria activity. Interestingly, plasma malate was decreased after SARS‐CoV‐2 infection.^18^ This observation suggests that mitochondria respiration is potentially suppressed due to deficient oxygenation in the pneumonia state.^19^ However, lactate, the glycolytic product produced under hypoxic conditions, did not show significant changes in SARS‐CoV‐2‐positive patients. Reduced oxygenation may have a minimal effect on glycolysis at least in serum metabolome.^18^ Notably, metabolites in the pentose phosphate pathway (PPP) showed a modest increase in COVID‐19 serum.^18^ PPP intermediate xylulose 5‐phosphate (X5P) was decreased in a different cohort of COVD19 patients, which could be explained by cohort‐specific variations.^20^

Alterations in amino acid metabolism are closely linked to the inflammatory and immune response in human body.^21^, ^22^ Kynurenine is a tryptophan‐derived amino acid and a key metabolite regulating inflammation and immunity. Circulating kynurenine was strongly increased in COVID‐19 patients and correlated with interleukin‐6 (IL6) levels.^18^ Circulating amino acids also serve as physiological indicators of organ functions. For instance, amino acids generated from the urea cycle, such as arginine, ornithine, and citrulline, were dysregulated in patient serum.^18^, ^23^ The nitrogen acceptor carbamoyl phosphate was also decreased.^20^ Both observations point to renal dysfunction in COVID‐19 patients.^18^ Gluconeogenic amino acids including alanine, glycine, and serine were increased, suggesting a systemic increase in energy requirement.^18^ Further, the decrease in sulfur‐containing amino acids such as cysteine and taurine potentially indicated SARS‐CoV‐2‐mediated oxidative stress.^18^ Noteworthy, specific amino acids or their derivatives correlated with COVID‐19 development.^24^ γ‐aminobutyric acid (GABA) is a neurotransmitter and immune‐modulatory metabolite.^25^ Downregulation of GABA correlated with disease severity and could be used for stratification of COVID‐19 patients.^26^

Serum lipid levels are intimately linked to COVID‐19 development. Both acylcarnitines and free fatty acids were increased in COVID‐19 patient serum.^18^ A remarkable disturbance of sphingolipid and arachidonic acid metabolism pathways was also observed.^26^ Notably, a percentage of COVID‐19 patients are asymptomatic and become hidden drivers of the pandemic. Serum metabolome profiling in asymptomatic patients revealed dysregulation of arachidonic acid metabolism, fatty acid β‐oxidation, and bile acid synthesis.^27^ As an immune‐modulatory metabolite, arachidonic acid may be utilized as a marker for the antiviral response during asymptomatic infection.

### 2.2 SARS‐CoV‐2 infection dysregulates blood cell metabolism

Circulating blood cells are implicated in SARS‐CoV‐2‐induced metabolic dysfunction (Figure 1). C‐reactive protein (CRP) is widely used as a serological inflammation marker.^28^ CRP levels were positively linked to glucose transporter 3 and monocarboxylate transporter SLC16A3 (solute carrier family 16 member 3) levels in blood cells,^29^ which indicated that glucose uptake was enhanced to maintain energy supply in COVID‐19 patients. Surprisingly, patients with high CRP showed decreased mitochondrial respiration and TCA cycle activity.^29^ These findings collectively suggest a reliance on glycolysis but not mitochondrial respiration for energy production in circulating blood cells.

Although red blood cells (RBCs) cannot support viral replication due to the lack of nucleus, angiotensin‐converting enzyme 2 (ACE2)‐interacting proteins were expressed on RBC membrane that potentially allows viral entry.^30^ Proteomic analysis of RBCs from COVID‐19 patients revealed an elevation of glycolytic enzyme phosphofructokinase, which was in accordance with an increase in glycolytic activity. Besides, short‐ and medium‐chain saturated fatty acids, acyl‐carnitines, and sphingolipids were decreased, indicating remodeling of lipid metabolism and cell membrane composition in RBCs. Importantly, antioxidant enzymes such as peroxiredoxin 1 (PRDX1), superoxide dismutase 1 (SOD1), and glucose‐6‐phosphate dehydrogenase (G6PD) were decreased. This is a possible outcome of oxidative damage‐induced protein degradation.^31^, ^32^

### 2.3 Metabolic disorder of intestinal function and microecology

Intestinal epithelium expresses viral receptor ACE2 and is therefore vulnerable to SARS‐CoV‐2 infection.^33^ Both intestinal function and microbiota metabolism were altered in COVID‐19 patients (Figure 1). Intestinal epithelium downregulated ACE2 expression after SARS‐CoV‐2 infection, leading to upregulation of sodium‐glucose cotransporter (SGLT).^34^ Because SGLT is responsible for glucose absorption from the blood, intestinal SARS‐CoV‐2 infection contributes to the onset of metabolic disorders in COVID‐19 patients. Additionally, SARS‐CoV‐2 evaded amino acid transportation by suppressing solute carrier family 6 member 19 (SLC6A19), the major luminal Na^+^‐dependent neutral amino acids transporter in the intestine.^35^ Spike protein recognized the ACE2–SLC6A19 complex and promoted its internalization. Consequently, neutral amino acid transportation was impaired.^36^ Notably, tryptophan and glutamine are major substrates of SLC6A19. Both amino acids play important role in cytokine release, tight junction formation and microbial defense.^37^ Disturbance of SLC6A19 activity by SARS‐CoV‐2 infection contributed to leaky gut and microbial dysbiosis, further exacerbating cytokine storm in COVID‐19 patients.^38^

SARS‐CoV‐2 targets intestinal microecology to induce inflammation.^39^ The diversity of fecal microbiota was significantly decreased in COVID‐19 patients.^40^ Genomic and metabolomic profiling of fecal samples revealed broad metabolic alterations between COVID‐19 patients and healthy controls.^41^ Strikingly, short‐chain fatty acid and isoleucine biosynthesis were suppressed ever after COVID‐19 recovery.^41^ SARS‐CoV‐2 infection possibly resulted in sustained metabolic damage to gut microbiome and intestine function. Consistently, fecal microbiomes of COVID‐19 patients were enriched with opportunistic pathogens.^42^, ^43^ Specific bacterial species, such as *Coprobacillus*, *Clostridium ramosum*, and *Clostridium hathewayi*, were found in positive correlation with the severity of SARS‐CoV‐2 infection. Meanwhile, *Faecalibacterium prausnitzii*, *Eubacterium rectale*, and bifidobacteria were downregulated or even absent in COVID‐19 patients.^44^ Fecal microbial markers might serve as a noninvasive diagnostic tool for COVID‐19.^39^

Imbalanced microecology promoted intestinal disease development, such as diarrhea and abdominal pain.^45^, ^46^ After clearance of SARS‐CoV‐2, more than half of patients suffered from post‐acute COVID‐19 syndrome.^47^, ^48^, ^49^, ^50^ Impaired microecology potentially deregulated intestinal metabolism and host immune response. Therefore, restoration of intestinal microecology may alleviate post‐acute COVID‐19 syndrome.

### 2.4 Metabolic effects of pancreatic and hepatic injury

Meta‐analysis of COVID‐19 serum showed that amylase and lipase were increased in a part of SARS‐CoV‐2‐positive patients, which correlated with worse clinical outcomes.^51^ This finding points to viral injury of the pancreas and liver^52^, ^53^ (Figure 1). Pancreatic and hepatic injuries may further cause metabolic complications that deteriorate clinical outcomes, as diabetic patients suffered more from lung damage in COVID‐19 cases.^54^

SARS‐CoV‐2 infection resulted in long‐term hyperglycemia in nearly a third of individuals who are normoglycemic before infection.^55^ Accordingly, clinic follow‐up showed that SARS‐CoV‐2 infection caused new‐onset diabetes or aggravation of pre‐existing metabolic dysfunction including obesity, hypertension, ketoacidosis, nonalcoholic fatty liver diseases, and diabetes.^34^ The increase in glucose levels in COVID‐19 patients is at least in part due to impaired pancreatic function. Pancreatic beta cells express ACE2 receptor and its associating proteins that allow SARS‐CoV‐2 infection.^56^ Coronavirus‐like particles were recently found in autophagolysosomes of pancreatic acinar cells of SARS‐CoV‐2 patients. These particles potentially impaired pancreatic islet function by altering the expression and distribution of host proteins.^57^ In addition, an abnormal serum cytokine profile was reported to cause insulin resistance and β cell hyperstimulation in patients without diabetic history.^55^

Liver injury represents another feature of SARS‐CoV‐2 infection. Approximately 15–53% of SARS‐CoV‐2 patients developed clinical features and pathological changes resembling hepatic injury.^58^, ^59^ ACE2 is overexpressed in both parenchyma hepatic cells and nonparenchyma hepatic cells.^60^ SARS‐CoV‐2 could bind to ACE2 protein expressed at hepatocytes, bile duct cells, and hepatic endothelial cells to induce metabolic reprogramming or even apoptosis. However, which cell types serve as the major targets of SARS‐CoV‐2 under physiological conditions remains to be elucidated.^61^ Studies using cultured liver cancer cell lines suggested that viral spike (S) protein could bind to cholesterol.^62^ Further, high‐density lipoprotein (HDL) scavenger receptor B type 1 (SR‐B1) mediated SARS‐CoV‐2 attachment and entry.^63^, ^64^ During this process, HDL enhanced cell surface binding of SARS‐CoV‐2 S protein.^63^ This observation explains why high cholesterol presented as a risk factor for severe COVID‐19 diseases.^65^ Importantly, COVID‐19 autopsy studies revealed dysregulated glucose and fatty acid metabolism in the liver.^66^ Specifically, glycogenolysis, galactose degradation, and glycolysis were suppressed while fatty acid oxidation and oxidative phosphorylation were activated in COVID‐19 cases.^66^ These data indicate that SARS‐CoV‐2 proteins may rewire specific metabolic pathways in human liver during its lifecycle.

Although the exact role of liver injury in COVID‐19 development remained under discussion,^67^ viral infection‐induced elevation of aspartate aminotransferase (AST) and alanine aminotransferase (ALT) was positively relevant to disease severity.^68^ Patients with hepatocellular carcinoma or those who underwent liver transplantation exhibited worse outcomes after SARS‐CoV‐2 infection.^69^ Dysregulation of liver metabolism potentially contributed to the complications observed in severe COVID‐19 patients.^66^, ^68^, ^70^, ^71^, ^72^

### 2.5 Arrogating adipose tissue

Obesity has been recognized as a high‐risk factor in COVID‐19 patients. Patients with metabolic syndrome are prone to develop more severe diseases upon exposure to SARS‐CoV‐2.^73^ Mechanistic studies suggest that both cell state and nutrition status are key determinants of ACE2 levels in host adipocytes.^34^ ACE2 expression was induced during the in vitro differentiation of adipocytes, implying that SARS‐CoV‐2 only targeted adipocytes in their mature state.^74^ Additionally, the lipid‐rich microenvironment where adipocytes lived promoted ACE2 expression.^75^ Rich lipid storage within adipocytes not only facilitated lipid raft formation on the cell membrane that perquisites viral entry, but also provided building blocks for viral capsules^54^ (Figure 1). Consequently, SARS‐CoV‐2 arrogated adipocytes for rapid replication and expansion, leading to adipose tissue dysfunction and insulin resistance.^74^ Inhibition of lipase‐mediated lipid breakdown strongly suppressed viral propagation in adipocytes.^76^ Interestingly, SARS‐CoV‐2 was found in the adipose tissue of overweight males but not in females.^76^

### 2.6 Destabilizing immunometabolism

SARS‐CoV‐2‐induced destabilization of immunometabolism is reflected in serum metabolome (Figure 1). Kynurenine metabolism belongs to one of the most prominent pathways that regulate host inflammatory events.^76^ Plasmic kynurenic acid (KA) and its derivatives were proposed to be markers for viral infection.^77^, ^78^ In addition to serological changes, SARS‐CoV‐2 mediated specific immunometabolic responses in host T cells. Effector T cells and memory T cells are vital for immune response and viral clearance. While effector T cells have enhanced glycolysis, memory T cells rely on oxidative phosphorylation and fatty acid oxidation for energy production.^79^ In COVID‐19 patients, a unique population of T cells was characterized by overexpression of voltage‐dependent anion channel 1 and translocase of outer mitochondrial membrane. Both proteins reside in mitochondrial membrane and sustain cellular respiration. Yet, mitochondria in these lymphocytes have misshaped cristae and deficient respiration.^80^ The aberrant mitochondrial morphology explained a preapoptotic phenotype of this population and dysfunctional immune response in COVID‐19 patients.

Aged patients usually went through rapid and progressive deterioration of COVID‐19.^34^ Coronavirus infection models established with aged mice phenocopied human SARS‐CoV‐2 infection and revealed immunometabolic alterations. A glycolysis‐to‐ketolysis switch protected host animals against inflammatory damage after murine beta coronavirus (mCoV) infection. Interestingly, a ketogenic diet promoted the expansion of protective γδ T cells and alleviated inflammation in mCoV‐infected aged mice.^81^ Ketone metabolism may be beneficial in the immune defense against SARS‐CoV‐2 infection.

## 3 HOST METABOLISM MODULATES SARS‐COV‐2 LIFECYCLE

**FIGURE 2:**
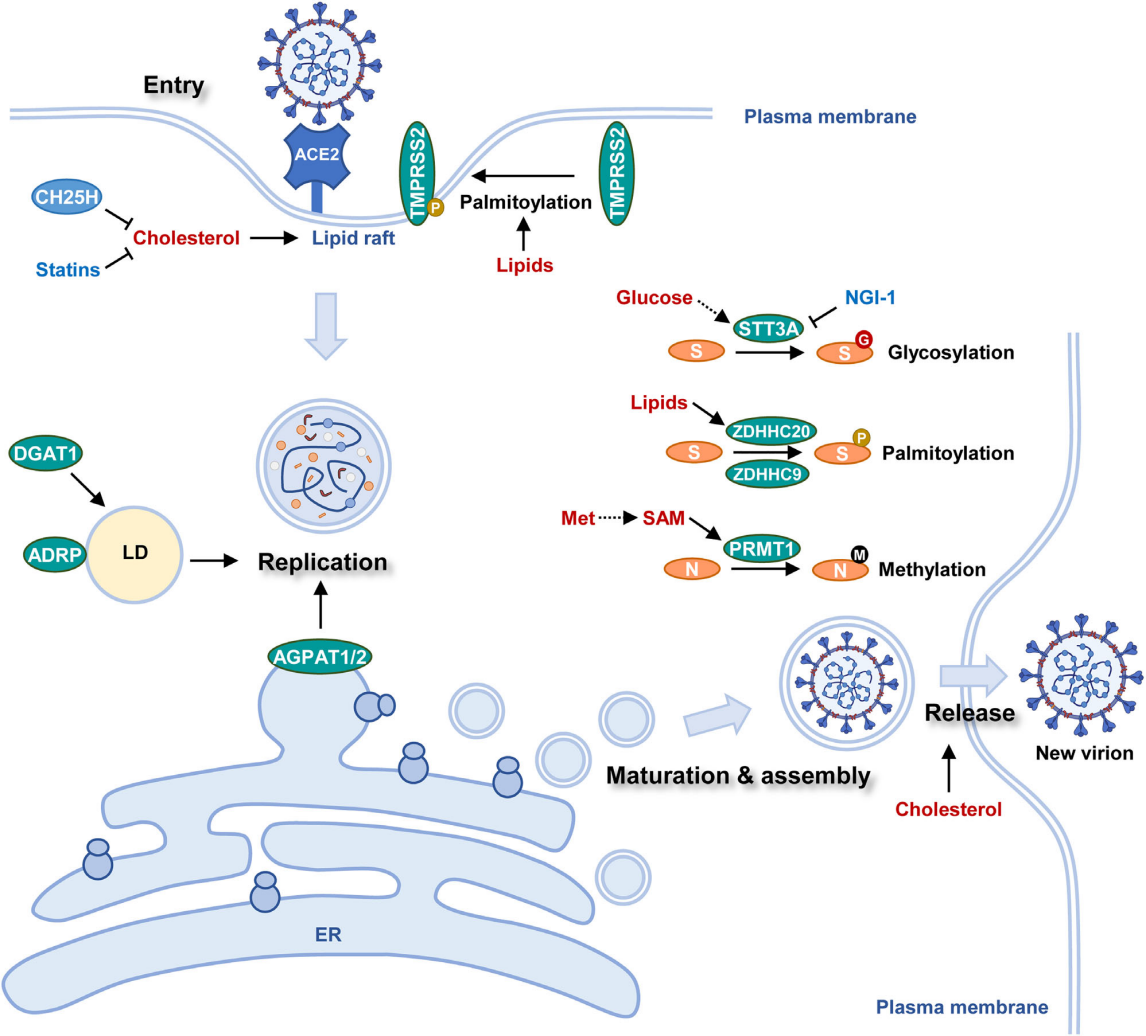
Host metabolism modulates SARS‐CoV‐2 lifecycle. SARS‐CoV‐2 lifecycle intensively interacts with host metabolism. Viral entry, replication, assembly, and release are modulated by host metabolism. Host cells provide metabolic support for SARS‐CoV‐2 biogenesis and maturation of viral proteins. ACE2 (angiotensin‐converting enzyme 2), TMPRSS2 (transmembrane serine protease 2), CH25H (cholesterol 25‐hydroxylase), DGAT1 (diacylglycerol O‐acyltransferase 1), ADRP (adipose differentiation‐related protein), LD (lipid droplet), AGPAT1/2 (1‐acylglycerol‐3‐phosphate O‐acyltransferase 1/3), ER (endoplasmic reticulum), S (spike protein), STT3A (STT3 oligosaccharyltransferase complex catalytic subunit A), ZDHHC20 (zinc finger DHHC‐type palmitoyltransferase 20), ZDHHC9 (zinc finger DHHC‐type palmitoyltransferase 9), Met (methionine), SAM (S‐adenosylmethionine), PRMT1 (protein arginine methyltransferase 1), N (Nucleocapsid protein),

SARS‐CoV‐2 lifecycle comprises at least four stages: entry, replication, assembly, and release^82^ (Figure 2). Multiple types of viruses, including SARS‐CoV‐2, human immunodeficiency virus, and influenza virus, use lipid rafts on host cell membrane as an entry point of the infection cycle. Receptor proteins and endocytosis machinery usually coexist in lipid rafts. SARS‐CoV‐2 infection starts from the interaction between viral S protein and ACE2 (Figure 2). Further, transmembrane serine protease 2 (TMPRSS2), which locates in proximity to ACE2, primes viral infection through enzymatic cleavage of S protein.^83^ The ACE2‐containing lipid raft mediates the internalization of virions to form endosomes.^84^ Subsequent proteolytic cleavage of S protein by cysteine proteases in late endosome or lysosome allows the release of viral nucleocapsid into host cytoplasm.^85^ After that, the virus replicates in double‐membrane vesicles (DMVs). DMVs are structurally similar to autophagosomes. Nonstructural proteins induce the formation of DMVs, of which cholesterol‐rich sites support viral replication.^86^ Therefore, cholesterol metabolism supports both viral entry and replication.^87^ Viral genome‐encoded structural and accessory proteins are synthesized by the host ribosomes at the endoplasmic reticulum (ER). These proteins are subsequently transferred to the Golgi apparatus where they will be assembled with viral genome, encapsulated into vesicles, and released into extracellular space via exocytosis.^88^, ^89^ Noteworthy, host metabolic network intertwines with both antiviral signaling and viral biogenesis to modulate SARS‐CoV‐2 lifecycle. Host cells provide essential building blocks for the biogenesis of viral genomes, proteins, and lipid membranes. Recent studies have identified key metabolic events that support viral amplification.

### 3.1 Lipid and cholesterol metabolism regulates SARS‐COV‐2 entry

Cholesterol is a key component of lipid raft and provides structural support for the ACE2 receptors. Upon SARS‐CoV‐2 infection, interferon (IFN) induces cholesterol 25‐hydroxylase (CH25H), an ER membrane‐bound protein. CH25H converts cholesterol into 25‐hydrocholesterol (25HC) and suppresses viral infection through two different mechanisms: (1) CH25H activation depletes cholesterol on the plasma membrane to destabilize ACE2 receptor‐containing lipid raft; (2) 25HC generated by CH25H activates acyl‐CoA:cholesterol acyltransferase on the ER, leading to internalization of cholesterol on the plasma membrane, further decreasing its cholesterol content (Figure 2). Both actions impeded viral entry and conferred a broad antiviral activity to 25HC.^90^

Interestingly, the importance of cholesterol in COVID‐19 development was firstly noticed in clinical records of hospitalization. Patients pretreated with statins, a blood cholesterol‐lowering drug, before hospital admission generally had reduced severity and morbidity.^91^ Hence, cholesterol‐reducing agents hold great potential to suppress viral infection (Figure 2). At least three different strategies were proposed to inhibit cholesterol metabolism^92^: (1) Sequestrants‐induced removal of cholesterol. Sequestrants such as β‐cyclodextrin could absorb cholesterol‐containing bile acids and sterols to deplete plasma cholesterol^93^; (2) Activation of cholesterol efflux. Compounds such as LXR agonists upregulate the expression of ATP‐binding cassette transporters A1 and G1, further promoting cholesterol secretion. Besides, cholesterol carriers or acceptors such as HDL or apolipoprotein A‐I could be employed to reduce cholesterol levels in circulation; (3) Inhibition of cholesterol synthesis. Chemical inhibitors of cholesterol synthesis other than statins are potently effective against viral infection.

Viral entry is also modulated by lipid metabolism. Lipid species not only support lipid raft formation as structural components but also serve as precursors for post‐translational modification (PTM). Fatty acylation is a PTM process during which fatty acids, such as palmitate, are attached to cysteine, serine, or threonine residues of proteins.^94^ Due to its hydrophobic nature, the palmitoyl chain enhances the affinity between modified proteins and lipid membranes.^95^ TMPRSS2, the viral entry priming protease, is at least in part regulated by palmitoylation^96^ (Figure 2). Increased lipid levels in obese patients may result in TMPRSS2 hyper‐palmitoylation and enhance its membrane localization, explaining the SARS‐CoV‐2 vulnerability in overweight individuals.

### 3.2 Lipid droplets support viral replication

After entering into cells, SARS‐CoV‐2 genome and proteins exist in DMVs and further hijack host metabolism for the biogenesis of new viral nucleotides and proteins.^97^ DMVs are centers for viral replication where the replication and transcription complexes anchor. The biogenesis of DMVs depends on host lipid phosphatidic acid (PA). Acylglycerolphosphate acyltransferase (AGPAT) 1 and 2 in the ER accelerated viral replication by increasing PA synthesis.^98^ Unexpectedly, lipid droplets (LDs) were recently found to be uncanonical sites for viral expansion. LDs are lipid‐rich organelles with monolayered membranes. Monocytes from COVID‐19 patients accumulated LDs compared with their counterparts from healthy individuals. Strikingly, SARS‐CoV‐2 proteins and double‐stranded RNA were located in close proximity to LDs. Electron microscopy of infected cells uncovered the colocalization of viral particles and LDs.^99^ LDs potentially served as a replicating center and assembly platform for SARS‐CoV‐2. Diacylglycerol O‐acyltransferase 1 (DGAT1) is the key enzyme responsible for triacylglycerol synthesis and LD formation. Besides, adipocyte differentiation‐related protein (ADRP) associates with LDs and maintains their structure. Depletion of either gene effectively downregulated SARS‐CoV‐2 amplification^100^ (Figure 2).

### 3.3 Assembly and maturation of SARS‐CoV‐2 rely on host metabolism

Viral proteins undergo a series of PTMs before they are assembled into new virions. Host cells provide metabolic precursors for PTM of viral proteins. The membrane‐bound S protein of SARS‐CoV‐2 was sequentially modified by lipid species with the help of zinc finger DHHC‐type palmitoyltransferase 20 and 9 (ZDHHC20 and ZDHHC9)^101^ (Figure 2). Notably, S protein acylation was indispensable for its maturation and viral budding at the Golgi apparatus. Besides, S protein acylation increased the fusion capacity of SARS‐CoV‐2 and thus enhanced its infectivity. Inhibition of protein acylation is a promising strategy to restrict viral infection.^101^

Sugar groups contribute to viral protein maturation in the form of glycosylation. Mass spectrometry studies uncovered that the majority of SARS‐CoV‐2 encoded proteins were glycosylated.^102^ SARS‐CoV‐2 employed host glycosylation enzymes, in particular STT3 oligosaccharyltransferase complex catalytic subunit A (STT3A), to modify S proteins (Figure 2). Once conjugated on spike protein, the sugar groups shielded the viral particle from immunosurveillance.^102^, ^103^ Chemical inhibition of spike protein glycosylation by NGI‐1 greatly weakened viral infectivity.^104^ Animal studies also supported the important role of lipids and sugars in COVID‐19 development. After a continuous high‐fat high‐sugar diet, SARS‐CoV‐2‐infected Syrian hamsters showed increased weight loss and lung dysfunction, delayed viral clearance, and prolonged viral shedding.^105^ Therefore, restriction of dietary lipid and sugar intake is potentially beneficial for COVID‐19 patients who have obesity or metabolic syndrome.

PTMs are also found on the nucleocapsid (N) protein. The N protein complexes with both SARS‐CoV‐2 genome and viral membrane proteins during virion assembly. Arginine residues (R95 and R177) in the N protein were methylated by protein arginine methyltransferase 1 (PRMT1) in the host cells. Nucleocapsid protein methylation promoted viral packaging^106^ (Figure 2). Chemical inhibition of PRMT1 downregulated N protein methylation and impaired SARS‐CoV‐2 replication.^106^ Arginine methylation level is regulated by the availability of S‐adenosyl methionine (SAM), the methyl donor produced from methionine.^107^ Therefore, methionine metabolism potentially modulates viral assembly and packaging (Figure 2).

### 3.4 Lipid raft modulates SARS‐CoV‐2 transmission

Mature SARS‐CoV‐2 virions are released into extracellular space through membrane rearrangements. Interestingly, both SARS‐CoV and SARS‐CoV‐2 can avoid exposure to extracellular environment through direct cell‐to‐cell transmission. Intercellular channels or syncytia facilitated the uncanonical transfer of the virus.^108^ Notably, lipid raft was involved in the formation of intercellular channeling tubes and syncytia. Thus, targeting lipid rafts suppressed both viral infection and transmission^109^ (Figure 2).

## 4 MECHANISMS OF REMODELING HOST METABOLISM BY SARS‐COV‐2

**FIGURE 3:**
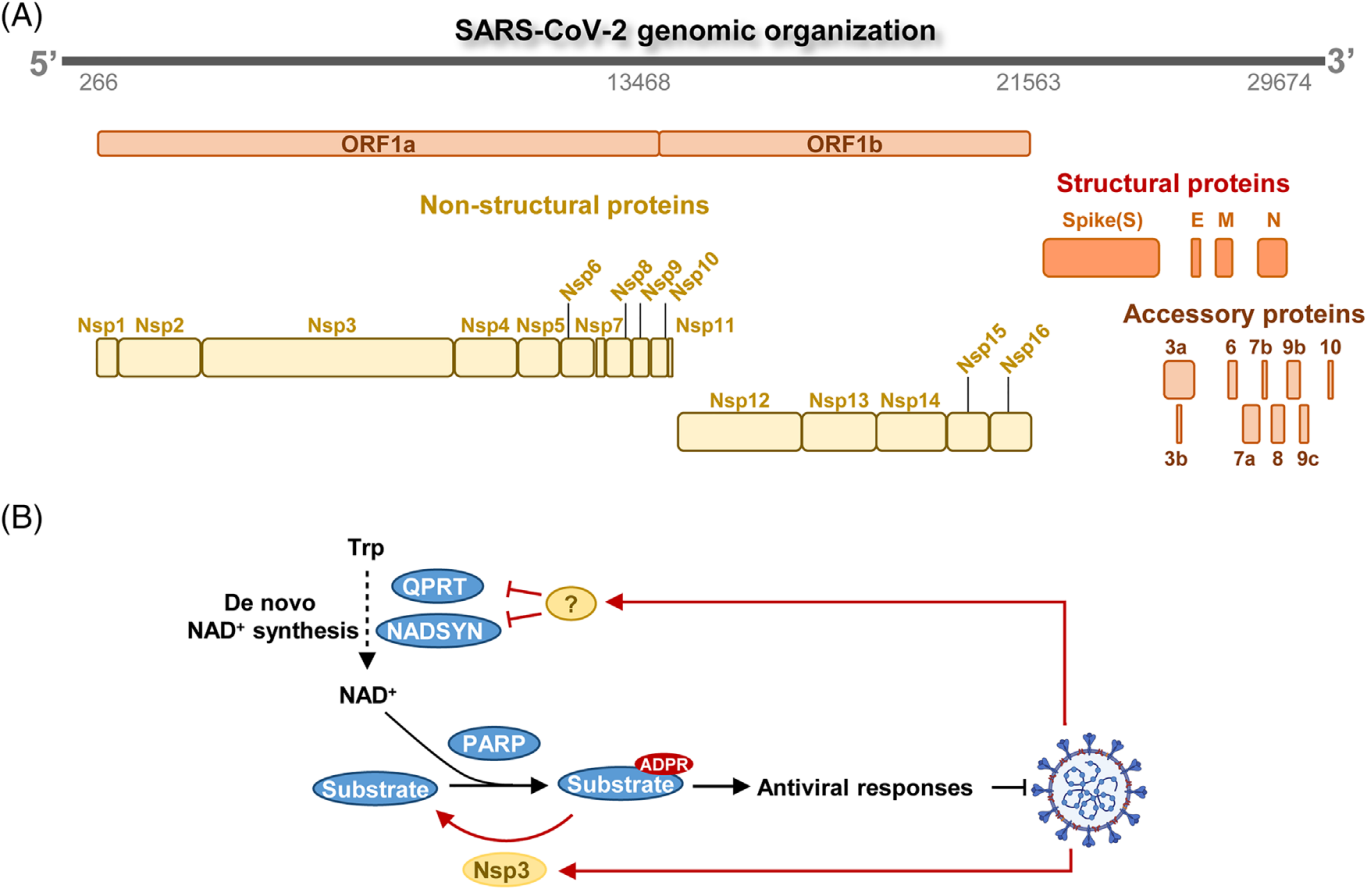
Mechanisms of remodeling host metabolism by SARS‐CoV‐2. (A) Organization of SARS‐CoV‐2 genome. SARS‐CoV‐2 genome encodes nonstructural proteins (Nsps), structural proteins, and accessory proteins. Structural proteins include Spike protein (S), Envelope protein (E), Membrane protein (M), and Nucleocapsid protein (N). (B) SARS‐CoV‐2 reshapes NAD^+^ metabolism to suppress antiviral response. SARS‐CoV‐2 protein Nsp3 counteracts with PARP to suppress ADPR modification and antiviral responses. Unknown viral factor(s) suppresses de novo NAD^+^ biosynthesis, which also inhibits PARP‐induced ADP‐ribosylation (ADPR) modification of substrate protein.

The metabolic changes in host cells could be outcomes of either host antiviral response or SARS‐CoV‐2‐induced metabolic remodeling. SARS‐CoV‐2 genome has a compact size and consists of ∼30,000 nucleotides. Proteins encoded by the viral genome fall into three categories: (1) four structural proteins including spike protein (S), envelope protein (E), membrane protein (M), and nucleocapsid protein (N); (2) 16 nonstructural proteins (nsp1‐16); and (3) accessory proteins including ORF3a, ORF3b, ORF6, ORF7a, ORF7b, ORF8, ORF9b, ORF9c, and ORF10^110^ (Figure 3A). Viral factors remodel host metabolism, which at least partially phenocopies reprogrammed cancer metabolism. In cancer cells, oncogenic mutations rewire metabolic networks to sustain malignant proliferation.^111^ Viral genome and its products likewise manipulate host cell metabolism to ensure rapid amplification of SARS‐CoV‐2. In agreement with enhanced biosynthetic needs, SARS‐CoV‐2 replication is dependent on anabolic signaling pathways such as phosphatidylinositol 3‐kinase/protein kinase B/mammalian target of rapamycin signaling.^112^ Although our knowledge remains in its infancy, multiomic profiling of virus–host interaction has shed light on how SARS‐CoV‐2 remodels host metabolism.

### 4.1 SARS‐CoV‐2 proteins disturb NAD^+^ metabolism to suppress antiviral response

NAD^+^ metabolism is perhaps one of the best studied metabolic pathways during SARS‐CoV‐2 infection (Figure 3B). Intracellular NAD^+^ pool is maintained by tryptophan‐dependent de novo NAD^+^ synthesis and salvage NAD^+^ biosynthesis. NAD^+^ serves as a donor for ADP‐ribose units and is required by poly (ADP‐ribose) polymerase (PARP) to mediate ADP‐ribosylation (ADPR) of antiviral proteins (Figure 3B). Host cells overexpress PARP and activate antiviral proteins by upregulating their ADP‐ribosylation.^113^, ^114^ Activation of PARP quickly exhausts intracellular NAD^+^. Notably, SARS‐CoV‐2 adopts at least two different strategies to suppress antiviral responses. First, viral protein Nsp3 is an ADPR hydrolase that removes ADPR modification to counteract host PARPs^115^ (Figure 3B). Second, SARS‐CoV‐2 restricts NAD^+^ supply with unknown factors to suppress antiviral signaling. Specifically, quinolinate phosphoribosyl transferase (QPRT) and NAD^+^ synthetase (NADSYN), the enzymes responsible for de novo NAD^+^ synthesis, were decreased by CoV infection (Figure 3B). Host cells could partially compensate for NAD^+^ shortage by enhancing salvage NAD^+^ biosynthesis. Importantly, chemical activation of NAD^+^ biosynthesis repressed viral infection and replication.^116^

### 4.2 Virus–host interactome reveals metabolic targets of SARA‐CoV‐2

**FIGURE 4:**
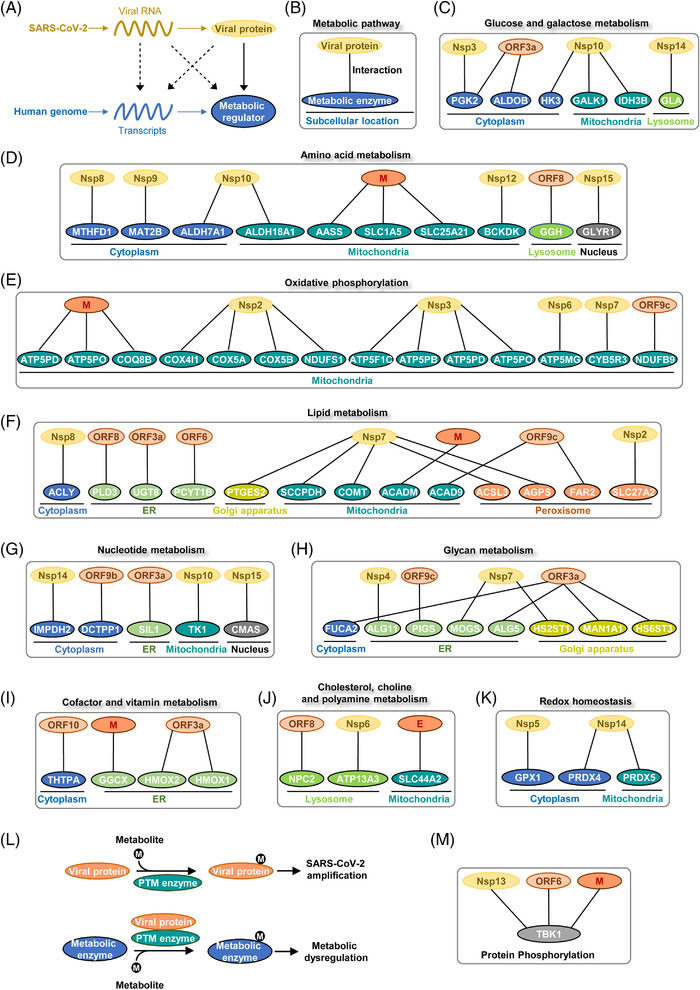
Protein–protein interactions between human and SARS‐CoV‐2. (A) Models showing how viral genome products (RNAs, proteins) interact with host metabolic networks. Protein–protein interactions between SARS‐CoV2 and humans have been elucidated in proteomic profiling studies. (B) Proteomic profiling of SARS‐CoV‐2 interactome revealed the physical association between viral proteins and host metabolic enzymes. The protein–protein interactions are categorized by metabolic pathways and subcellular location. (C) Viral proteins interacted with host enzymes involved in glucose and galactose metabolism. (D) Physical interactions between viral proteins and host enzymes in amino acid metabolism. (E) Viral proteins interacted with host proteins involved in oxidative phosphorylation. (F) Physical associations between viral proteins and host enzymes in lipid metabolism. (G) Viral proteins interacted with host proteins involved in nucleotide metabolism. (H) Interactions between viral proteins and host enzymes involved in glycan metabolism. (I) Viral proteins interacted with host enzymes involved in cofactor and vitamin metabolism. (J) Viral proteins were associated with host enzymes involved in cholesterol, choline and polyamine metabolism. (K) Viral proteins interacted with host enzymes involved in redox homeostasis. (L) Different modes of the interaction between host metabolism, PTM enzyme, and viral infection. Viral proteins may serve as either substrates or modulators of PTM enzymes to interact with host metabolic networks. (M) The physical association of host TBK1 with viral proteins was used as an example to explain virus–host metabolism interaction.

SARS‐CoV‐2 genome and its products potentially modulate host metabolism at multiple levels such as transcription, translation, and PTM of metabolic regulators^14^, ^63^, ^117^, ^118^, ^119^, ^120^, ^121^, ^122^, ^123^ (Figure 4A). Mechanisms underlying how viral proteins hijack host cell metabolism remain largely unclear. Protein–protein interactions provide a molecular basis for SARS‐CoV‐2‐mediated metabolic remodeling. To comprehensively illustrate the metabolic interaction between SARS‐CoV‐2 and human body, we extracted the interactions between viral proteins and host metabolic enzymes from published proteomic studies^14^, ^123^ and grouped them by metabolic pathways and subcellular compartments (Figure 4B). Unexpectedly, viral proteins interacted with multiple central pathways in host cell metabolism, such as sugar metabolism, amino acid metabolism, lipid metabolism, nucleotide metabolism, and redox homeostasis. Another surprising observation from protein interaction studies is that viral proteins interacted with metabolic enzymes from different subcellular locations. Viral proteins might enter into different organelles to rewire metabolic pathways in a compartmentalized manner. Of note, phosphoglycerate kinase 2 (PGK2), fructose‐bisphosphate aldolase b (ALDOB), and hexokinase 3 (HK3) are cytosolic enzymes involved in glycolysis.^124^, ^125^, ^126^ Interaction with viral proteins may impact the enzymatic activity or stability of these enzymes, resulting in dysregulation of glucose metabolism in host cells upon SARS‐CoV‐2 infection (Figure 4C). G6PD catalyzes the rate‐limiting step of the PPP, which provides NADPH and pentose phosphates for fatty acid and nucleic acid synthesis.^127^ Individuals with G6PD deficiency suffer a hemolytic crisis when exposed to oxidants or microbes. They are supposed to be more susceptible to SARA‐CoV‐2 infection.^128^, ^129^, ^130^

SARS‐CoV‐2 proteins have broad interactions with transporters and enzymes from amino acid metabolism. For instance, M protein was associated with transporter solute carrier family 1 member 5 (SLC1A5), transporter solute carrier family 25 member 21 (SLC25A21), and lysine degradation enzyme aminoadipate‐semialdehyde synthase (AASS)^131^, ^132^, ^133^ (Figure 4D). Notably, SARS‐CoV‐2 protein may be able to sense the metabolic status of host cells. Viral protein Nsp12 belongs to RNA‐dependent RNA polymerases. Nsp12 contains a nucleotidyltransferase domain distant from the catalytic center. This domain functions as a nucleotide sensor and activates RNA synthesis in the presence of nucleotides.^134^, ^135^ Hence, Nsp12 may sense host nucleotide availability and correspondingly modulates replicative efficacy of viral genome. Interestingly, Nsp12 interacted with branched‐chain keto acid dehydrogenase kinase, a mitochondrial enzyme responsible for branched‐chain amino acid (BCAA) catabolism^136^ (Figure 4D). Two cofactors of Nsp12, Nsp7, and Nsp8, interacted with the electron transport chain (ETC) and ribosome proteins in mitochondria^137^ (Figure 4E). These observations indicate that BCAA metabolism and mitochondrial respiration are potentially under the control of viral proteins.

Lipid metabolism and nucleotide biosynthesis are reprogrammed during SARS‐CoV‐2 infection.^100^ Enzymes from both fatty acid beta‐oxidation (acyl‐CoA dehydrogenases, ACADM and ACAD9) and lipogenesis (ATP citrate lyase, ACLY) were found to interact with viral proteins.^138^, ^139^, ^140^ Strikingly, Nsp7 seems to be a potent regulator of lipid metabolism as it interacted with four different enzymes from this pathway. Among binding partners of Nsp7, acyl‐CoA synthetase long‐chain family member 3 (ACSL3) converts free long‐chain fatty acids into fatty acyl‐CoA esters to support lipid biosynthesis^141^ (Figure 4F). Intriguingly, ACSL3 has been found to maintain the replication of different viruses and serves as a promising therapeutic target for COVID‐19.^142^ In accordance, SARS‐CoV‐2 infection reduced ATP production and deregulated fatty acid metabolism in a cell‐based infection model.^143^ Unknown virulent factors from SARS‐CoV‐2 upregulated lipid uptake and lipogenesis genes in monocytes.^99^ DGAT and ADRP were also upregulated to promote the formation of LDs. Moreover, viral nucleocapsid protein interacted with ADRP on the LD surface to facilitate viral replication.^144^

Viral genome replication and transcription consume large amounts of nucleotides. Inosine monophosphate dehydrogenase 2 (IMPDH2) and thymidine kinase 1 (TK1) are rate‐limiting enzymes that control guanine and thymidine nucleotide biosynthesis, respectively.^145^, ^146^ Viral proteins may boost nucleotide biosynthesis by activating these enzymes (Figure 4G). Noteworthy, folate metabolism provides one‐carbon units to maintain nucleotide synthesis.^147^ SARS‐CoV‐2 infection resulted in folate metabolism remodeling to enhance de novo purine synthesis and viral genome replication.^148^

Glycans play vital roles in cellular structure maintenance, protein or lipid modification and cell‐to‐cell communications.^149^, ^150^ Importantly, ORF3a was associated with fucosidase 2 (FUCA2) which catalyzes the hydrolysis of carbohydrate moieties from glycoproteins. FUCA2 has been revealed as an oncogenic factor due to its immunosuppressive activity.^151^ Whether ORF3a induces host immunosuppression through interacting with FUCA2 still needs further validation (Figure 4H).

Cofactor and vitamin metabolism are candidates for viral targets as well. Heme metabolism end‐products, such as ferrous iron, biliverdin, and bilirubin, have antiviral effects depending on their antioxidant, anti‐inflammatory, and anti‐apoptotic functions.^152^, ^153^, ^154^, ^155^ Heme oxygenase (HMOX), an essential enzyme in heme catabolism, might be a therapeutic target for COVID‐19.^156^, ^157^ Viral ORF3a interacted with both isozymes, HMOX1 and HMOX2, implying a significant role of heme metabolism during SARS‐CoV‐2 infection (Figure 4I).

As mentioned earlier, cholesterol is essential for SARS‐CoV‐2 entry and replication. Once entered into cells, SARS‐CoV‐2 in turn upregulated host cholesterol metabolism by modulating SREBP2‐mediated gene expression.^158^ Viral proteins ORF8, Nsp6, and E interacted with enzymes in cholesterol, choline, and polyamine metabolism. The biological function of these interactions remains unclear (Figure 4J).

Redox imbalance represented a key metabolic outcome of COVID‐19.^36^, ^83^, ^159^, ^160^, ^161^ Redox homeostasis is involved in the whole lifecycle of SARS‐CoV‐2 and host inflammatory responses.^162^, ^163^, ^164^ Viral protein Nsp5 and Nsp14 interacted with redox‐regulatory enzymes in both cytoplasm and mitochondria (Figure 4K). SARS‐CoV‐2 may coordinate redox balance and biosynthetic processes in different cellular compartments to maximize its amplification efficacy. Metabolic interaction between SARS‐CoV‐2 and human hosts will provide more clues for therapeutic opportunities.

### 4.3 The effect of SARS‐CoV‐2 on mitochondrial function and metabolism

A remarkable number of metabolic enzymes from SARS‐CoV‐2 interactome reside in mitochondria, highlighting a vital role of mitochondria in viral lifecycle. Mitochondria generate ATP and metabolic intermediates to support the biosynthesis of macromolecules.^165^ Moreover, they function as hubs in cell metabolism, stress signaling and immune response.^166^, ^167^ Importantly, viral proteins bind to multiple enzymes residing in the mitochondria matrix (Figures 4C–4K). Although it remains unclear whether or not mitochondria serve as the replicating platform for SARS‐CoV‐2, accumulating evidence has shown that mitochondria function is broadly affected by SARS‐CoV‐2 infection.

SARS‐CoV‐2 remodels mitochondria metabolism after entering into host cells. In cancer cells, suppression of mitochondria activity drives the redistribution of carbon flux into glycolysis and PPP to satisfy anabolic needs.^32^, ^135^ Similarly, mitochondrial activity is suppressed in multiple models of viral infection.^168^ The interaction between viral proteins and oxidative phosphorylation implies that viral factors may directly modulate cellular respiration and carbon fluxes (Figure 4E). In support of this speculation, the transcriptional activity of nuclear factor erythroid 2‐related factor 2 (NRF2), a nuclear factor that drives mitochondrial biogenesis, was broadly suppressed in COVID‐19 patients' biopsies.^167^, ^169^, ^170^, ^171^ Chemical activation of NRF2 exerted antiviral effects toward SARS‐CoV‐2 infection.^168^ Of note, mouse hepatitis virus infection model suggested that endogenous metabolites could alter mitochondrial metabolism and correspondingly modulate viral replication efficacy.^172^ Thus, metabolic interventions may be employed to restrain mitochondria metabolism and SARS‐CoV‐2 replication.^172^, ^173^ It is worthy to mention that previous proteomic studies utilized overexpressed viral proteins as baits to track virus–host interaction.^63^, ^123^ Further efforts are required to exclude potential experimental artifacts in physical association with mitochondrial enzymes. Which viral factors function as drivers of metabolic remodeling remains an open biological question.

Mitochondria‐derived reactive oxygen species (ROS) act as metabolic signals that modulate SARS‐CoV‐2 amplification. Mitochondria utilize ETC to fulfill oxidative phosphorylation and energy production (Figure 4E). Leakage of electrons from the ETC results in the production of ROS, making mitochondria a major source of oxidative stress. SARS‐CoV‐2 infection triggered mitochondrial ROS accumulation to interrupt host antiviral responses. In COVID‐19 patients, elevated mitochondrial ROS associated with mitochondrial membrane depolarization.^174^ Specifically, ORF3a caused mitochondrial damage and elevated mitochondrial ROS to upregulate hypoxia‐inducible factor 1α (HIF‐1α). HIF‐1α  further induced proinflammatory responses to enhance viral infection.^175^ In monocytes, elevated mitochondrial ROS stabilized HIF‐1α and promoted glycolysis. Dysregulation of monocyte metabolism inhibited T cell activity and reduced lung epithelial cell viability.^176^ In a complementary study, cluster of differentiation 147 (CD147, also known as basigin) was found to strongly bind to S protein of SARS‐CoV‐2. The consequent ROS accumulation contributed to metabolic dysfunction of T cells and impaired virus clearance. Nutrients such as fatty acid and glucose were proposed to alleviate mitochondrial ROS production in T cells to fight against viral infection.^177^, ^178^

SARS‐CoV‐2 also affects mitochondrial mass and organelle dynamics. Mitochondria maintain organelle‐specific genomes (mtDNA). Both bulk and single‐cell RNA sequencing of COVID‐19 blood samples showed that mtDNA‐encoded gene expression levels were decreased. This phenomenon is possibly resulted from reduced expression of mitochondrial RNA polymerase.^179^, ^180^ Unexpectedly, T cells, monocytes, and granulocytes were reported to have increased mitochondrial mass. However, only T cells exhibited higher oxidative phosphorylation capacity and architecture disruption.^178^ Besides, hepatocytes from the liver samples of SARS‐CoV‐2‐positive patients were also characterized by cristae disruption.^181^ Mechanistically, ORF9b promoted mitochondrial elongation by proteasomal degradation of dynamin‐related protein 1 (DRP1), a protein responsible for membrane remodeling during mitochondrial division.^182^ Viral proteins ORF9c, M, Nsp6, and ORF3a were shown to localize to mitochondrial membrane and induce morphological changes.^183^ Of note, viral proteins encoded by SARS‐CoV‐2 genome were reported to interact with multiple proteins located in mitochondrial inner membrane. Mitochondrial permeability transition pore (mPTP) is a protein complex formed in the inner membrane under specific pathological conditions.^184^ The opening of mPTP increases mitochondria permeability and leads to mitochondrial swelling.^185^ Viral proteins may hamper mitochondrial function through binding to mPTP and destructing mitochondrial inner membrane integrity. Depolarization of mitochondria could further impair mitophagy and cause apoptosis.^174^, ^183^, ^186^, ^187^ Notably, mPTP blocker cyclosporin A was demonstrated to protect cardiomyocytes from SARS‐CoV‐2‐induced autophagy and cell death.^183^

The cytokines released during SARS‐CoV‐2 infection are critical determinants of mitochondrial function and metabolism.^188^ IFN‐γ induced by viral infection acted on Toll‐like receptor signaling in macrophages and initiated mitochondria‐driven cell death.^189^, ^190^ In this process, nitric oxide synthase was induced to promote caspase‐8 cleavage and activate mitochondrial apoptotic effectors. Importantly, the mitochondria‐driven apoptosis potentially increased disease severity in COVID‐19 patients.^189^ In critically ill COVID‐19 cases, both pro‐inflammatory (IL‐1, IL‐2, IL‐6, TNF‐α) and anti‐inflammatory (IL‐4, IL‐10) cytokines were presented at high levels.^191^ The metabolic influences of these cytokines on mitochondrial function remains vague. Previous studies have shown that IL‐1 suppressed mitochondrial fatty acid oxidation to drive tissue‐specific inflammation.^192^, ^193^ IL‐4 was reported to promote mitochondrial glutaminolysis and α‐ketoglutarate production in macrophages.^194^

### 4.4 The interaction between PTM enzymes and host metabolism during SARS‐CoV‐2 infection

PTMs of viral proteins represent a key regulatory mechanism of SARS‐CoV‐2 lifecycle.^195^ In addition to known modifications on the spike and nucleocapsid proteins (Figure 2), a variety of PTMs presumably exists on SARS‐CoV‐2 proteome. Previous studies of human and animal coronavirus have revealed numerous PTM events on viral proteins, such as glycosylation, phosphorylation, ADP‐ribosylation, SUMOylation, and ubiquitination.^195^, ^196^ Noteworthy, SARS‐CoV‐2 proteins interacted with posttranslational modifying enzymes involved in protein methylation, phosphorylation, palmitoylation, and glycosylation.^14^ Because metabolic intermediates serve as substrates in the majority of PTM reactions,^197^, ^198^ cell metabolism has an intimate relationship with PTM of viral proteins.^199^, ^200^, ^201^ For example, protein kinase transfers phosphate group from ATP to modify its substrate protein in the form of phosphorylation. From this perspective, PTM enzymes potentially connect metabolic status to their downstream proteins. Given the fact that PTMs on spike and nucleocapsid proteins contribute to viral protein maturation and assembly, it would not be surprising that host metabolism is possibly involved in the regulation of other steps in SARS‐CoV‐2 lifecycle. The PTMs of viral proteins potentially interconnect host metabolic network with viral lifecycle to coordinate the replication and dissemination of viral particles. Therefore, PTMs may act as metabolic checkpoints of viral lifecycle and pave the way for metabolic intervention of COVID‐19. Additionally, metabolites are emerging as signaling molecules that regulate cellular biogenesis.^202^ Whether SARS‐CoV‐2 senses specific metabolic signals to prime viral replication remains an unsettled question. Proteomic profiling of SARS‐CoV‐2 proteins at different time points of infection would help to elucidate the dynamics of viral protein modification.

Viral proteins could act as either substrates or regulators of host PTM enzymes (Figure 4L). During SARS‐CoV‐2 infection, PTM enzymes are potential regulatory nodes that connect host metabolism and viral proteins (Figure 4L). Of note, TANK‐binding kinase 1 (TBK1) is a vital regulator of innate immunity and antiviral response. Previously, viral proteins Nsp13, ORF6, and M were shown to associate with TBK1.^14^ Through phosphorylating target proteins, TBK1 not only facilitated the activation of NF‐κB pathway but also promoted the expression of IFN‐stimulated genes.^203^, ^204^, ^205^, ^206^ Notably, Nsp13 and M protein promoted TBK1 degradation via autophagic process or ubiquitination, resulting in inhibition of host innate immune response^207^ (Figure 4M). Besides, TBK1 was previously reported to modulate energy metabolism by suppressing adenosine 5‘‐monophosphate‐activated protein kinase.^208^, ^209^ It is reasonable to speculate that other PTM enzymes may be involved in viral infection through interconnecting cell metabolism and SARS‐CoV‐2.

## 5 DISCOVERY OF METABOLISM‐BASED THERAPY TO COMBAT SARS‐COV‐2

The deepening knowledge of COVID‐19 metabolism enables the discovery of new therapeutic targets. Clinical evidence combined with genetic or chemical screening has revealed several metabolic vulnerabilities of SARS‐CoV‐2. Metabolism‐based therapy would provide powerful expansion to the arsenal against SARS‐CoV‐2.

### 5.1 Targeting cholesterol metabolism: lessons from human genetics

**FIGURE 5:**
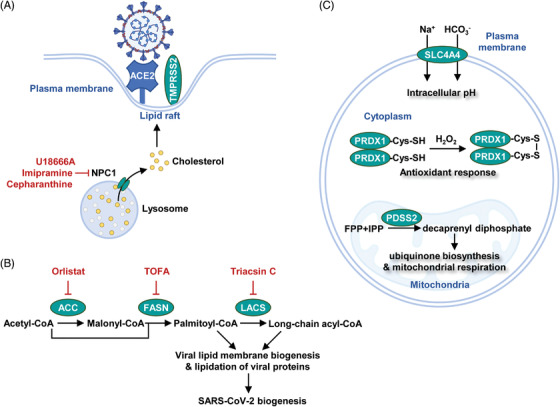
Discovery of metabolism‐based therapy to combat SARS‐CoV‐2. (A) NPC1, a lysosomal protein, mediated cholesterol efflux to promote the formation of lipid rafts on the plasma membrane. Chemical inhibition of NPC1 suppressed lipid raft‐induced viral entry. (B) Lipid synthesis supported SARS‐CoV‐2 biogenesis. Chemical inhibition of lipogenic enzymes, such as ACC (acetyl‐CoA carboxylase), FASN (fatty acid synthase), and LACS (long‐chain acyl‐CoA synthetases), suppressed viral replication. (C) Metabolic dependencies identified by high‐throughput screening. SLC4A4 (solute carrier family 4 member 4), PRDX1 (peroxiredoxin 1), and PDSS2 (decaprenyl diphosphate synthase subunit 2) were identified to be necessary metabolic factors for SARS‐CoV‐2 infection

Human genetic diversity provides insights into disease progression and treatment.^210^ In the case of COVID‐19, genetic evidence highlights the role of Niemann‐Pick disease type C1 (NPC1) gene in viral infection. NPC1 locates at the lysosomal membrane and mediates cholesterol efflux^211^ (Figure 5A). Inherent mutations of this gene sequester cholesterol in the lysosome and lead to dysregulation of lysosomal proteolytic activity. As proteolysis of spike proteins is an essential step for viral infection, NPC1 mutations conferred a broad resistance against filovirus, retrovirus, and togavirus.^212^ Homozygous deletion of *Npc1* also protected mice from viral infection,^212^ making NPC1 an exploitable target for COVID‐19 treatment.^213^ Small molecule inhibitors of NPC1, such as U18666A, showed a broad antiviral activity including coronavirus.^214^, ^215^ Of note, imipramine and cepharanthine are FDA‐approved drugs targeting the NPC1 pathway. Both compounds reduced the infectivity of several single‐stranded RNA viruses^216^, ^217^ (Figure 5A).

In addition, human genome‐wide association studies identified a link between solute carrier family transporter 6A20 (SLC6A20) with interstitial pneumonia in COVID‐19 patients.^218^ SLC6A20 locates on the plasma membrane and mediates proline/glycine uptake in a calcium‐dependent manner. Further investigations are required to pinpoint the therapeutic opportunities behind SLC6A20.

### 5.2 Lipid and glucose metabolism as feasible therapeutic targets

Clinical observations in hospitalized COVID‐19 patients revealed a link between statin use with improved outcomes.^219^, ^220^ Statin potentially slowed COVD‐19 pathogenesis by suppressing lipid metabolism. However, concerns were also raised because statin increased the expression of ACE2 receptor. Alternative inhibitors of lipid biosynthesis showed promising activity against SARS‐CoV‐2. For example, inhibitors of fatty acid synthase (orlistat), long‐chain acyl‐CoA synthetases (triacsin C), and acetyl‐CoA carboxylase (TOFA) effectively suppressed virus infection (Figure 5B). In contrast, lipid β‐oxidation seems to play a minor role in virus replication.^221^ Interestingly, polyunsaturated fatty acids weakened viral infection. Linolenic acid and eicosapentaenoic acid (EPA) suppressed TMPRSS2 protease to block SARS‐CoV‐2 entry.^222^

Diabetic COVID‐19 patients exhibited lower mortality if prescribed glucose‐lowering drugs.^223^, ^224^ The virulence of SARS‐CoV‐2 may be determined by environmental factors such as glucose concentration in the tissue microenvironment. The efficacy of glucose‐lowering drugs in nondiabetic patients remains unclear.

### 5.3 Genome‐wide screening of essential metabolic genes for viral infection

Genome‐wide CRISPR screening serves as a powerful tool for identifying key factors for viral infection.^225^ In monkey kidney cells, metabolic genes including solute carrier family transporter 4A4 (SLC4A4, a bicarbonate cotransporter and pH modulator), PRDX1 (a peroxide‐detoxifying enzyme), and decaprenyl diphosphate synthase subunit 2 (PDSS2, an enzyme in ubiquinone biosynthesis), were found to be essential for viral entry and replication^226^ (Figure 5C). Hence, cellular acid‐base balance, redox signaling, and cellular respiration potently govern SARS‐CoV‐2 infectivity. Compounds targeting these metabolic processes may be translated into COVID‐19 therapies.

### 5.4 Clinical trials of metabolic intervention of COVID‐19

After years of drug discovery, remdesivir is still one of the most effective drugs recommended for SARS‐CoV‐2 treatment.^227^, ^228^, ^229^ Remdesivir inhibits the RNA‐dependent RNA polymerase encoded by the viral genome to impair RNA synthesis and SARS‐CoV‐2 replication.^230^ Compared to compound screening from scratch, repurposing clinically approved drugs is more economic.^231^ Chemical screening in the field of cancer metabolism has generated a rich number of drugs that suppress various metabolic pathways.^232^, ^233^ Because SARS‐CoV‐2 reprograms host cell metabolism in a fashion similar to cancer cells. The preclinical drugs targeting cancer metabolism may be directly tested for antiviral activity. Indeed, bioactive molecules targeting metabolic enzymes in lipid metabolism and protein glycosylation were found to inhibit SARS‐CoV‐2 infection.^142^ Several metabolites from commensal microbiota were predicted to bind to SARS‐CoV‐2 spike, awaiting experimental validation.^234^

Importantly, several antiviral compounds suppress different metabolic pathways and are under clinical evaluation (Table 1). Palmitoylethanolamide (PEA) is a lipid‐derived peroxisome proliferator‐activated receptor‐α (PPAR‐α) agonist that prevents SARS‐CoV‐2 entry and replication.^235^ PEA was found to promote β‐oxidation and LD degradation.^235^ Baricitinib, an inhibitor of the Janus‐associated kinases 1 and 2 (JAK1 and JAK2), was demonstrated to modulate fatty acid oxidation in SARS‐CoV‐2 infected patients.^236^ Fenofibrate is a cholesterol‐lowering drug that holds potential to treat COVID‐19.^237^

**TABLE 1:** Clinical trials of metabolic intervention of COVID‐19

Drug	Metabolic target	Clinical trial number	Phase	Status	Drug type
PEA^235^	Lipid metabolism (PPAR‐α)	NCT04619706	2	Terminated	Chemical
		NCT04568876	4	Completed	
Pioglitazone^249^	Lipid metabolism (PPAR‐γ)	NCT04535700	4	Recruiting	Chemical
Baricitinib^236^	Fatty acid metabolism (JAK1/JAK2)	NCT04421027	3	Completed	Chemical
Fenofibrate^237^	Cholesterol metabolism	NCT04517396	2	Completed	Chemical
VPA^250^	Glucose metabolism (HDAC)	NCT04513314	4	Not yet recruiting	Chemical
Masitinib^240^	Potentially glucose metabolism (Tyrosine kinase)	NCT05047783	2	Recruiting	Chemical
Methotrexate^251^	Folate metabolism	NCT04610567	1	Recruiting	Chemical
Cholecalciferol^252^	Steroid metabolism	NCT04952857	4	Completed	Chemical
Nicotinamide^253^	Nicotinamide metabolism	NCT04751604	NA	Active, not recruiting	Chemical
L‐citrulline^254^	Amino acid metabolism	NCT04404426	NA	Completed	Chemical
Coenzyme Q10^255^	Mitochondrial metabolism	NCT04960215	2	Completed	Chemical

In addition to lipid metabolism, glucose metabolism is emerging as one of the major targets for antiviral drugs (Table 1). Valproic acid (VPA), a histone deacetylase (HDAC) inhibitor, was reported to lower glucose levels and inhibit cytokine response. Both actions may contribute to its inhibitory effect on SARS‐CoV‐2 entry.^238^ Masitinib, a tyrosine kinase inhibitor, inactivates viral protease to restrict viral replication. Although Masitinib has a significant effect on body glucose metabolism, its exact metabolic target is still unknown.^239^, ^240^ Of note, folate metabolism provides one‐carbon units to fuel nucleotide biosynthesis.^241^ Methotrexate suppresses folate metabolism and has been used for the treatment of multiple cancers. The efficacy of methotrexate in COVID‐19 development is under investigation in a clinical trial.^242^

## 6 DISCUSSION

SARS‐CoV‐2‐induced metabolic reprogramming plays a key role in COVID‐19 progression. Due to the limitation of animal models that recapitulate human COVID‐19 development, the mechanism of how SARS‐CoV‐2 rewires host metabolism remains poorly understood. Besides, SARS‐CoV‐2 is rapidly evolving and accumulating mutations worldwide. Newly occurring mutations confer new traits to SARS‐CoV‐2 and breach immune protection. Whether newly evolved SARS‐CoV‐2 variants remodel host metabolic in specific manners requires further investigation. Metabolic vulnerabilities shared by major SARS‐CoV‐2 strains will open the window to the discovery of broad‐spectrum antiviral compounds. Meanwhile, strain‐specific remodeling of host metabolic pathway creates the opportunity for precise and personalized treatment of COVID‐19.

### 6.1 Systemic profiling of metabolic interactions between host and SARS‐CoV‐2

SARS‐CoV‐2 was reported to cause long‐term metabolic complications in infected individuals.^243^ The impact of viral genome and its products on host cell metabolism remains largely unclear. It is necessary to evaluate the influence of each viral protein and RNA on host metabolic networks, such as mRNA expression, protein level, and catalytic activity of rate‐limiting enzymes. Besides, SARS‐CoV‐2‐mediated metabolic alterations vary among different tissues. In‐depth profiling of tissue‐specific metabolic reprogramming is essential to resolving COVID‐19 pathology. Of note, SARS‐CoV‐2 replication produces viral subgenome.^13^ Genomic and subgenomic RNAs are potentially neglected regulators of host metabolism.

### 6.2 Humanized animal model for SARS‐CoV‐2 infection

The lack of humanized SARS‐CoV‐2 infection model remains a bottleneck in COVID‐19 studies. Recently, a lung organoid model indicated that SARS‐CoV‐2 infection downregulated lipid metabolism, urea cycle, folate metabolism, and glutamine metabolism.^244^ Mouse models have been widely used to study coronavirus infection, but they could not faithfully reproduce the antiviral responses in humans.^245^ The development of humanized animal models for COVID‐19 would undoubtedly accelerate vaccine development and drug screening.^246^

### 6.3 Emerging mutations and new challenges

The new SARS‐CoV‐2 variant Omicron has exacerbated the global COVID‐19 pandemic. Novel COVID‐19 mutations mainly occur in the receptor‐binding domain of the spike protein.^247^ Whether these mutations rewire unique metabolic pathways in host cells remains unknown. It is thus important to track the metabolic changes caused by different variants, and correspondingly identify metabolic determinants their infectivity, virulence, and transmissibility.^248^ These efforts would give rise to predesigned therapies and enable our chase to be faster than SARS‐CoV‐2 mutations.^248^

## CONFLICT OF INTEREST

The authors declare no conflict of interest.

## ETHICS STATEMENT

Not applicable.

## AUTHOR CONTRIBUTIONS

T. W., Q. Z., and Y. P. W. conceptualized the work. T. W., Y. C., H. Z., Z. W., C. H. M., Y. Y., L. C., S. X., X. Y., Q. Z., and Y. P. W. wrote the paper. T. W., Q. Z., and Y. P. W prepared figures and table; T. W., Q. Z., and Y. P. W. supervised the work and revised the manuscript. T. W., Y. C., and H. Z. contributed equally to this work.

## Data Availability

Not applicable.
